# A novel deep learning-based 3D cell segmentation framework for future image-based disease detection

**DOI:** 10.1038/s41598-021-04048-3

**Published:** 2022-01-10

**Authors:** Andong Wang, Qi Zhang, Yang Han, Sean Megason, Sahand Hormoz, Kishore R. Mosaliganti, Jacqueline C. K. Lam, Victor O. K. Li

**Affiliations:** 1 Department of Electrical and Electronic Engineering, The University of Hong Kong, Hong Kong, China; 2 Department of Systems Biology, Harvard Medical School, Boston, MA USA

**Keywords:** Computational biology and bioinformatics, Image processing, Machine learning

## Abstract

Cell segmentation plays a crucial role in understanding, diagnosing, and treating diseases. Despite the recent success of deep learning-based cell segmentation methods, it remains challenging to accurately segment densely packed cells in 3D cell membrane images. Existing approaches also require fine-tuning multiple manually selected hyperparameters on the new datasets. We develop a deep learning-based 3D cell segmentation pipeline, 3DCellSeg, to address these challenges. Compared to the existing methods, our approach carries the following novelties: (1) a robust two-stage pipeline, requiring only one hyperparameter; (2) a light-weight deep convolutional neural network (3DCellSegNet) to efficiently output voxel-wise masks; (3) a custom loss function (3DCellSeg Loss) to tackle the clumped cell problem; and (4) an efficient touching area-based clustering algorithm (TASCAN) to separate 3D cells from the foreground masks. Cell segmentation experiments conducted on four different cell datasets show that 3DCellSeg outperforms the baseline models on the ATAS (plant), HMS (animal), and LRP (plant) datasets with an overall accuracy of 95.6%, 76.4%, and 74.7%, respectively, while achieving an accuracy comparable to the baselines on the Ovules (plant) dataset with an overall accuracy of 82.2%. Ablation studies show that the individual improvements in accuracy is attributable to 3DCellSegNet, 3DCellSeg Loss, and TASCAN, with the 3DCellSeg demonstrating robustness across different datasets and cell shapes. Our results suggest that 3DCellSeg can serve a powerful biomedical and clinical tool, such as histo-pathological image analysis, for cancer diagnosis and grading.

## Introduction

Computer-aided digital pathology plays an increasingly important role in understanding, diagnosing, and treating various kinds of diseases^1^. Digital image processing has become increasingly widespread in biological research as high-throughput/high-content microscopy and screening generate significant quantities of complex fine-grained cellular images^2^. With the development of modern biomedical techniques—such as cellular staining, whole-slide imaging, telemedicine, and cloud storage—millions of tissue biopsies are analysed annually^3,4^. To increase the efficiency of screening big cell image data, and to standardize image analysis and reduce variation in interpretability, there is a need to develop improved computer vision techniques for big cell image data analysis.

Cell segmentation plays a key role in biological image processing. Computer-aided diagnostics requires the identification of single cells. With regards to histo-pathological image analysis for cancer diagnosis and grading, the regularity of cell borders, shapes, and distributions provides an important insight into whether tissue regions are cancerous^5^. Other recent research studies have shown that the distribution and quality of blood cells are connected with the pathogenesis of Alzheimer’s Disease and may contribute to disease progression^6^. Cell segmentation has also been applied in studying the dynamics of gene regulation, cell growth and proliferation. Time-lapse microscopy technologies—including confocal, two photon, and light sheet microscopy—enable detailed data analytics based on dynamic cellular processes at the single-cell level^7,8^. However, recognizing cells as the objects of an image, and tracking these objects from one image to the next, still presents a central challenge^8^. The development of computational cell segmentation methods dramatically decreases time and labour in related biomedical applications.

Cell segmentation algorithms can be categorized into semantic segmentation and instance segmentation. Semantic segmentation refers to the partitioning of images into different semantic parts and assigning each pixel to a class (e.g. cell foreground or background). Instance segmentation seeks to identify each instance of the same class, by separately detecting and delineating every single cell shown in the image. Table 1 overviews the current deep learning-based methods for cell segmentation and lists their major drawbacks. For a more detailed description of traditional methods and deep learning-based approaches for cell instance segmentation, please refer to Supplementary Information.

**Table 1:** An overview of the existing deep learning models for 2D/3D cell segmentation.

Segmentation type		Deep learning model for 2D cell segmentation	Deep learning model for 3D cell segmentation	Major drawback
Semantic segmentation		U-Net^9^; DeepCell^10^	3D U-net^11^; V-Net^12^; VoxRexNet^13^; 3D-DSN^14^; 2D-3D^15^; C2FNAS^16^; Automatic Data Augmentation^17^	Fails to distinguish different cell instances
Instance segmentation	Contour-aware approach	DCAN^18^; Deep Watershed^19^	U-Net + CRF^20^; U-Net + SWS^21^; PlantSeg^22^; DISCo^23^; U-Net + Graph based^24^	Performance is highly dependent on the manually selected parameters during the post-processing procedures Prone to fuse cells that are tightly adhered
	Object-detection-based	Retinanet^25^; R-CNN, and a series of revised structures^26^–^28^; Keypoint bounding box^29^; PointINS^30^; FCOS^31^; CenterMask^32^; YOLACT^33^	Retina-Unet^34^; Weak Annotation^35^	Suffers from a severe imbalance between the number of positive and negative anchor boxes May fail to discern objects that are poorly approximated with bounding boxes
	Other strategies	GAN^36^; Embedding^37,38^; StarDist^39^; TensorMask^40^; AdaptIS^41^; CondInst^42^	StarDist 3D^43^; ShapeMetrics^44^; Spherical Harmonics^45^	Less accurate than the previous two mainstream strategies Many of these models are based on specific assumptions The training process of GAN networks is highly complex, especially on 3D datasets

Most existing 3D segmentation deep learning models focus on semantic segmentation. 3D U-Net^11^ extends 2D U-Net into 3D, incorporating a path for extracting high-level features and a path for generating segmented cells in full-resolution. V-Net is another 3D version of U-Net, with residual connections added between the convolutional layers^12^. VoxResNet extends a 2D deep residual network to a 3D residual network^13^. 3D-DSN is a 3D fully convolutional network equipped with a deep supervision mechanism^14^. These methods have been applied in various biomedical segmentation tasks, including image segmentation for brain, liver, prostate, and heart tissues. C2FNAS (coarse-to-fine neural architecture search)^16^ automatically identifies a 3D segmentation network, and^17^ employs automatic data augmentation for medical image segmentation tasks. Both of these two models^16,16^ have achieved state-of-the-art performance.

Some recent methods have applied instance segmentation on 3D cellular images^21–23,25,40,41^. These methods use deep learning based 3D semantic segmentation models in their first step to generate a pixel-based classification of the cell interiors, edges, and backgrounds. Traditional techniques, including thresholding and watershed, are then used to separate single cells from each other. Their major drawback is in processing clumped cells, which leads to the adhesion of cell instances after semantic segmentation, thereby degrading the segmentation accuracy. Furthermore, the performance of existing methods is highly dependent on the manually selected hyperparameters during the separation procedure. Many methods have deployed a large number of hyperparameters that are fine-tuned on pre-existing datasets, which require retuning on new datasets. Meanwhile, some models^58,59^ use shape priors to constrain model predictions to a set of natural variations, but these also require the cells to fall into a particular shape, thus making model generalization difficult. Instead of following the recent trends in developing more accurate and complex semantic segmentation models, we aim to build a simplified model of high robustness and efficiency, and yet achieving comparable performance in 3D cell segmentation.

**Figure 1:**
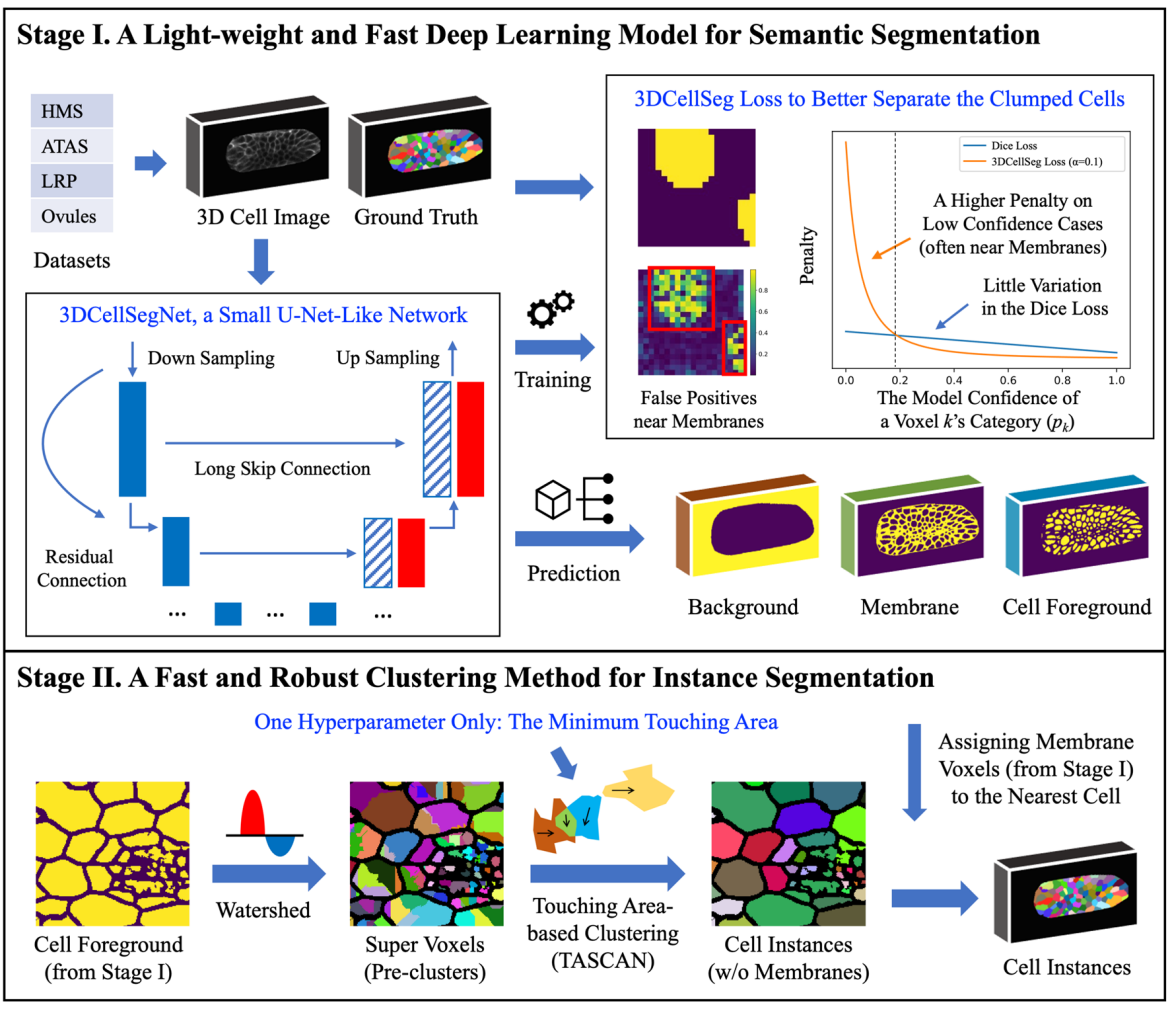
3DCellSeg: A two-stage light-weight, fast, and robust pipeline for 3D cell segmentation. [Note: There are two stages in the pipeline. The first stage is a semantic segmentation, where the input is a 3D cell membrane image and the output consists of three masks, which indicate whether a voxel is the cell foreground, membrane, or background. The second stage is an instance segmentation performed on the basis of these three masks. The cellular images and segmentation results were generated by Python Matplotlib (https://matplotlib.org) using the HMS dataset].

We propose 3DCellSeg, a novel domain-specific 3D cell instance segmentation model, with four distinctive novelties. A two-stage pipeline is followed. In the first stage, a light-weight CNN is developed to perform semantic segmentation. In the second stage, cell instance segmentation is conducted using a super voxel-based clustering algorithm (see Fig. 1).

The main novelties of 3DCellSeg are summarized in Table 2.

**Table 2:** Key novelties of 3DCellSeg.

Aspect	Novelty
Network	Based on the characteristics of cell membrane images, a light-weight network, 3DCellSeg, is designed to yield a fast inference speed while achieving an accuracy comparable or superior to the existing cutting-edge approaches
Loss function	A new loss function, 3DCellSeg Loss, is proposed to tackle the clumped cell problem
Post-processing	Inspired by DBSCAN (Density-based Spatial Clustering of Applications with Noise)^48^, a new clustering method, TASCAN (Touching Area-based Spatial Clustering of Applications with Noise) is proposed for 3D cell instance segmentation; TASCAN operates faster, achieves better performance, and requires only one single manually selected hyperparameter
Model usability	3DCellSeg pipeline is robust, easy to fine-tune, and outperforms existing cutting-edge methods across different experimental datasets

## Results

### 3DCellSeg pipeline

#### Semantic segmentation

Most existing biomedical image processing models, such as V-Net and VoxResNet, are designed for images containing multiple tissues and structures, such as CT or MRI images. However, the content of the cell membrane images is much simpler. We used this domain-specific knowledge to design our light-weight CNN model, 3DCellSegNet, which has a faster inference speed while achieving performance comparable to other state-of-the-art cell segmentation models.

#### Instance segmentation

Our TASCAN algorithm works on super voxels (small clusters of voxels). Only one hyperparameter in TASCAN (and for the whole pipeline) needs to be set: namely, the minimum touching area between two cell foreground super voxels. TASCAN helps reduce mis-clustering because the super voxels within the same cell usually have much larger touching areas than the super voxels across two neighboring cells. The super voxels across two neighboring cells are caused by membrane voxels being misclassified as the cell foreground, and this misclassified area is usually small in practice.

#### Loss function

Cells are densely packed across membrane images. Under existing methods, this results in the adhesion of cell masks after semantic segmentation, which greatly degrades the accuracy of instance segmentation. We therefore designed a new loss function, 3DCellSeg Loss, to address the problem. As the top right box in Fig. 1 shows, based on the Dice Loss function, 3DCellSeg adds weight matrices to penalize the voxels that are closer to the cell membranes and replaces $p_{k}$ (model confidence of a voxel being the cell foreground) with $\frac{{p_{k} }}{{p_{k} + \alpha }}$ (see Method for further details). This suppresses the segmentation of the cell foreground when the model confidence is low (which often happens near the cell membranes) and thus reduces adhesions of cell masks.

Four cell membrane image datasets consisting of both animal and plant tissues were used for training and testing: HMS (zebrafish cells); ATAS (Arabidopsis thaliana apical stem cells)^49^; LRP (Arabidopsis thaliana lateral root cells)^50^; and Ovules (Arabidopsis thaliana Ovules cells)^51^. Four types of metrics were used for evaluation: Jaccard Index (JI), Dice Similarity Coefficient (DSC), Adapted Rand Error (ARE)^52^, and Variation of Information (VOI)^53^. A detailed description of the experimental datasets and performance evaluation metrics can be found under Method.

#### Performance comparison

**Figure 2:**
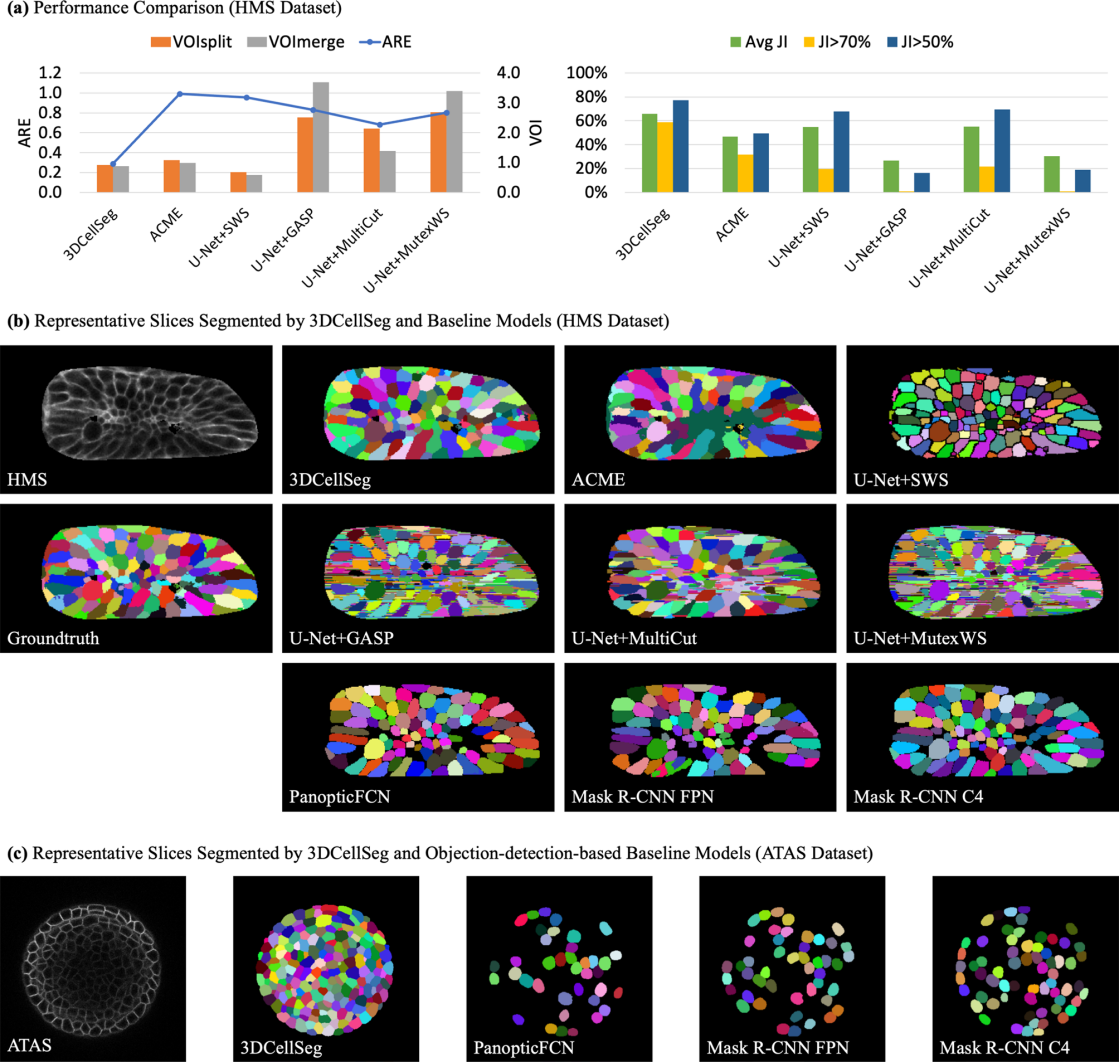
Model comparison and representative slices. [*Note* (**a**) shows the accuracies of different cell segmentation models for the HMS dataset. 3DCellSeg achieves the second best accuracy in ARE, VOI_split_, and VOI_merge_, and achieves the best accuracy in Avg JI, JI > 70%, and JI > 50% (the plots for DSC-related metrics are of high similarity to JI-related metrics). (**b**) and (**c**) show representative slices of different model segments. ACME tends to under-segment (see the dark green region which mis-classifies different cells as one cell) while U-Net + SWS tends to over-segment (see the over-segmented small cells in the central region). PanopticFCN, Mask R-CNN FPN, and Mask R-CNN C4 are accurate on the HMS dataset but they are severely under-segment on the ATAS dataset. The cellular images in (**b**) and (**c**) were generated by Python Matplotlib (https://matplotlib.org) using the HMS and ATAS^49^ datasets].

**Figure 3:**
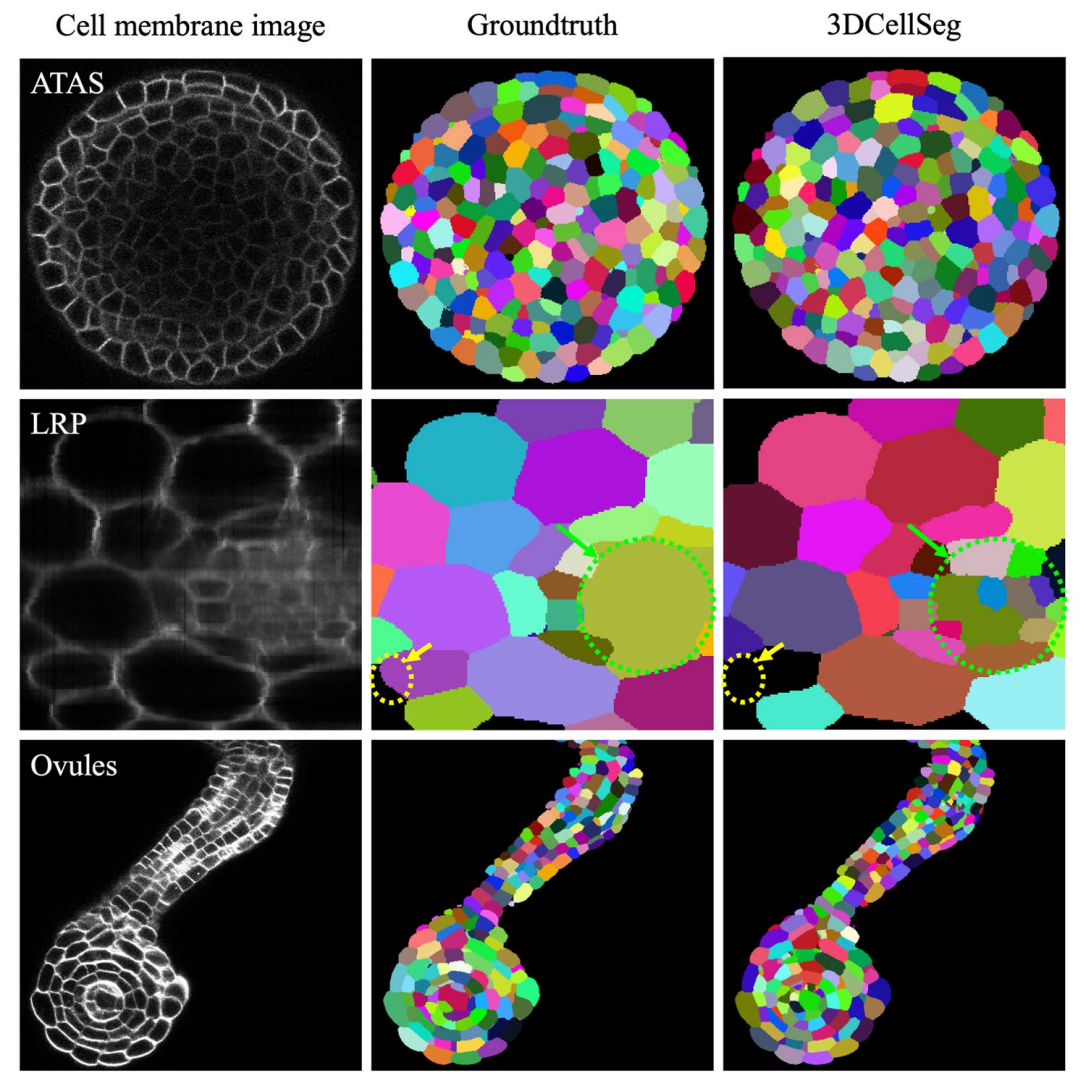
3DCellSeg performance on the ATAS, LRP, and Ovules datasets. [Note: Different cell instances were randomly assigned different colors. The LRP dataset images are annotated: the yellow circle shows where 3DCellSeg has made a mistake and the green circle shows that 3DCellSeg can segment cells that were not labelled in the ground truth. The cellular images were generated by Python Matplotlib (https://matplotlib.org) using the ATAS^49^, LRP^50^, and Ovules^51^ datasets].

We compared our 3DCellSeg with both traditional and deep learning methods. To benchmark 3D image performance, we compared 3DCellSeg with 3D U-Net based methods^11^. To test the model performance on the animal cell membrane images, we retrained U-Net + SWS^21^, U-Net + GASP^23,40^, U-Net + MultiCut^23,48^, and U-Net + MutexWS^23,41^ on the HMS dataset, and compared their performance with our method and ACME (traditional method, developed using the HMS dataset) (see Table 2 and Fig. 2). To ensure that our model comparison using the same set of plant cell membrane images is being fairly conducted, we compared our model with U-Net + SWS (built using ATAS) on the ATAS dataset, while U-Net + MutexWS, U-Net + MultiCut, and U-Net + GASP (built using LRP and Ovules) were compared with our method on the LRP and Ovules datasets (see Table 2 and Fig. 3). We used the default hyperparameters of other models (most of the hyperparameters relate to instance segmentation), and used the same hyperparameter (the minimum touching area between two cell foreground super voxels) for our model across the four datasets. We evaluated 3DCellSeg and other baseline models with nine metrics: ARE, VOI_split_, VOI_merge_, Avg JI, Avg DSC, JI > 70%, DSC > 70%, JI > 50%, and DSC > 50% (for their definitions, see Datasets and metrics in Method). The results are shown in Table 3. For ARE, VOIsplit, and VOImerge, the lower the value, the higher the accuracy. For Avg JI, Avg DSC, JI > 70%, DSC > 70%, JI > 50%, and DSC > 50%, a higher value indicates a higher accuracy.

**Table 3:**
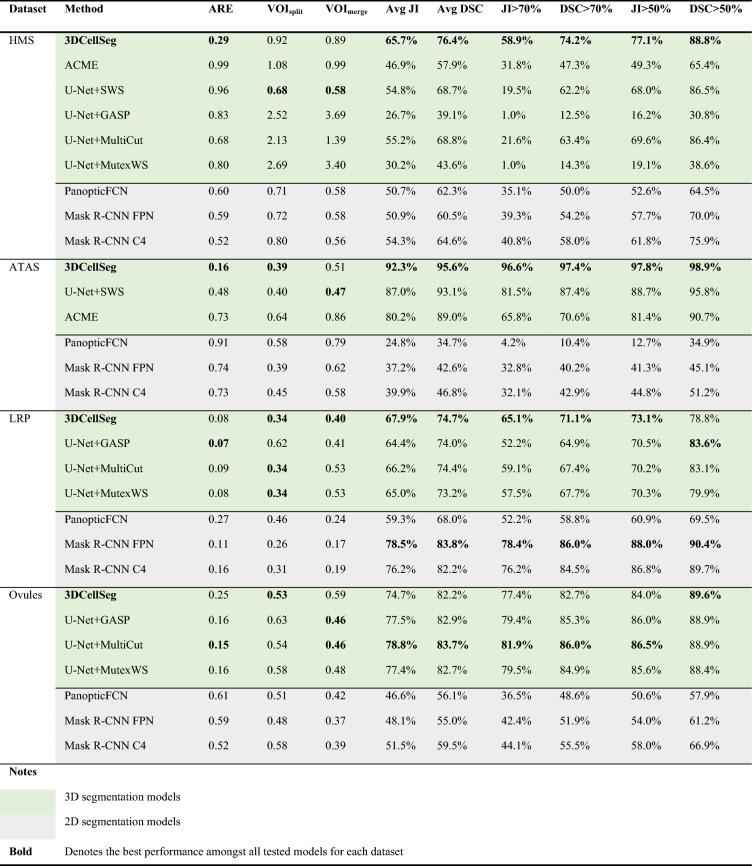
Comparison of model performance on the HMS, ATAS, LRP, and Ovules datasets.

[Note: For the HMS dataset, U-Net + SWS, U-Net + GASP, U-Net + MultiCut, and U-Net + MutexWS were retrained on default hyperparameters, and compared with our 3DCellSeg. For the ATAS dataset, U-Net + SWS, which was originally developed, trained and fine-tuned on the ATAS dataset, was compared with 3DCellSeg. For the LRP and Ovules datasets, U-Net + GASP, U-Net + MultiCut, and U-Net + MutexWS , which were originally built, trained and fine-tuned on LRP and Ovules, were compared with 3DCellSeg. Object-detection based instance segmentation methods (PanopticFCN, Mask R-CNN FPN, and Mask R-CNN C4) trained on 2D slices of the HMS, ATAS, LRP, and Ovules datasets were also taken as baselines for model comparison. ARE, VOI_split_, VOI_merge_, JI-related, and DSC-related metrics were calculated on 3D space for 3DCellSeg, ACME, U-Net + SWS, U-Net + GASP, U-Net + MultiCut, and U-Net + MutexWS and were calculated on 2D slices for PanopticFCN, Mask R-CNN FPN, and Mask R-CNN C4].

To assess 2D image performance, we ran three object-detection-based instance segmentation models developed for 2D images: two Mask R-CNN^28^ models with different backbones (ResNet-50-C4 and ResNet-50-FPN^55^, denoted as Mask R-CNN C4 and Mask R-CNN FPN respectively) and a currently-published panoptic segmentation method named PanopticFPN^56^. These models were fed 2D slices of cell membrane images from HMS, ATAS, LRP, and Ovules for model training and testing. ARE, VOI_split_, VOI_merge_, JI -related, and DSC-related metrics were calculated for each 2D segmentation method. The results are shown in Table 3. For all the results shown in this article (except for the ablation study for transfer learning in Table 4), the models were re-trained on the same dataset they were tested on. We observe that since segmenting 2D images is intrinsically easier than segmenting 3D images, if the accuracy values of the three object-detection-based models for 2D images are lower than those of 3DCellSeg and other baseline models for 3D images, then the performance deficit of the three object-detection-based models when adapted to segment 3D images will be even larger.

**Table 4:**
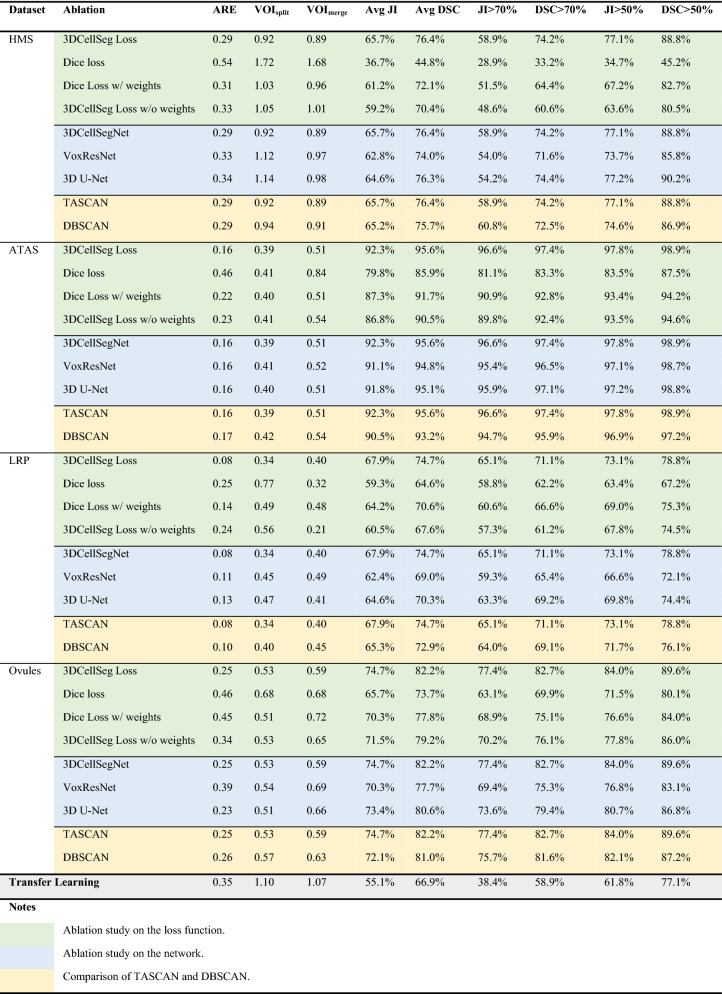
Ablation studies showing 3DCellSeg Loss, 3DCellSegNet, TASCAN, and Transfer Learning.

As shown in Table 3, 3DCellSeg achieves the best performance over three datasets, the HMS, ATAS, and LRP, while attaining a comparable performance on the Ovules dataset. U-Net + SWS, U-Net + GASP, U-Net + MultiCut, and U-Net + MutexWS were built and fine-tuned on Arabidopsis thaliana cell membrane images (the ATAS, LRP, and Ovules datasets). However, their performance drops significantly when applied to the zebra fish cell membrane images (the HMS dataset). It has been noted that U-Net + GASP and U-Net + MutexWS perform less satisfactorily as compared to the traditional ACME method on the HMS dataset. This might be related to their reliance on a significant number of manually selected hyperparameters, which are fine-tuned on the membrane images of a certain type of cells, and may not be transferrable to a new type of cells. 3DCellSeg is more robust, as it requires only one hyperparameter.

Moreover, though PanopticFCN, Mask R-CNN FPN, and Mask R-CNN C4 can achieve a high accuracy on the LRP dataset when only a few cell instances are shown on one 2D slice, they perform poorly on the HMS, ATAS, and Ovules datasets when many more cell instances (100–300) are shown on each slice. The results show that 3DCellSeg is more robust while object detection-based instance segmentation method may work much less satisfactorily when the number of target instances to be segmented is large.

Additionally, the size of the 3DCellSegNet (~ 5 MB) adopted in our pipeline is much smaller than the U-Net (~ 15 MB) in U-Net + SWS, and the U-Net (~ 50 MB) in U-Net + GASP, U-Net + MultiCut, and U-Net + MutexWS, with significant reductions in inference time (the inference time of 3DCellSeg is around 50% to 70% of that of other baseline models on the ATAS, LRP, and Ovules datasets).

## Ablation studies

Ablation studies show that instance segmentation accuracy is improved by our CNN model 3DCellSegNet, our loss function 3DCellSeg Loss, and our clustering algorithm TASCAN.

### Effects of 3DCellSeg loss

First, we evaluate the effect of the loss function. We compare our 3DCellSeg Loss with the original Dice Loss function (denoted as Dice Loss), Dice Loss with weight matrices (denoted as Dice Loss w/ weights), and 3DCellSeg Loss with the replacement $\frac{{p_{k} }}{{p_{k} + \alpha }}$ but without weight matrices (denoted as 3DCellSeg Loss w/o weights) on the four datasets (shown in the green-colored rows in Table 4). We find that weight matrices and replacement both greatly improve segmentation accuracy on all metrics. When both are incorporated into the model, the accuracy is further improved.

### Effects of 3DCellSegNet

Second, we compare the performance of 3DCellSegNet with that of the two commonly used models for medical image processing, VoxResNet and 3D U-Net, as the backbone CNN in our pipeline (shown in the blue-colored rows in Table 4). We find that all three models have a similar accuracy on the four datasets. However, 3DCellSegNet performs slightly better than the other two models on most metrics. With regards to model size and inference time, 3DCellSegNet (~ 5 MB) is much smaller than VoxResNet (~ 30 MB) and 3D U-Net (~ 50 MB). 3DCellSegNet takes less than 20 s to segment one membrane image from the HMS dataset while the other two require more than one minute.

### Effects of TASCAN

Third, we compare the accuracy of adopting TASCAN versus DBSCAN in instance segmentation in our pipeline on the four datasets. This is shown in the orange-colored rows in Table 4. TASCAN performs slightly better than DBSCAN. However, the execution time of TASCAN is less than 20 s while that of DBSCAN is about 180 s. The efficiency advantage of TASCAN becomes more apparent when tackling larger images because the execution time increases cubically with image size. Segmenting larger images like LRP and Ovules takes just a few minutes on TASCAN but more than one hour for a single image on DBSCAN.

### Transfer learning

The model’s transfer learning capability was tested. Based on 3DCellSegNet trained on ATAS dataset, we re-trained the models over only 2 images randomly-picked from the HMS training set. The last row in Table 4 showed that with transfer learning our pipeline still performed well over the HMS dataset, implying that our pipeline was robust and easily applicable to the new cell membrane datasets.

## Discussion

Most existing deep learning approaches to 3D cell segmentation follow a two-stage pipeline and focus on either CNN model architecture design in the semantic segmentation stage or post-processing design in the instance segmentation stage. However, the clumped cell problem in cell segmentation has not been adequately addressed, with challenges in accuracy and robustness as existing segmentation pipelines rely on extensive manually selected hyperparameters. To tackle these critical but overlooked problems, we propose a deep learning-based two-stage pipeline, 3DCellSeg. The whole pipeline requires only one manually selected hyperparameter (the minimum touching area between two super voxels of the cell foreground). In the first stage, a light-weight CNN-based U-Net-like model, 3DCellSegNet, performs semantic segmentation while addressing the clumped cell problem by incorporating a novel loss function, 3DCellSeg Loss, into the network training process. In the second stage, a novel touching area-based clustering algorithm, TASCAN, distinguishes cell instances from the other types of semantic voxels according to the minimum touching area.

The experimental results based on four animal and plant cell membrane image datasets show the performance of the 3DCellSeg pipeline to be better than or at least comparable to other approaches. For the HMS (animal), ATAS (plant), and LRP (plant) datasets, 3DCellSeg pipeline achieved better performance than the traditional baseline (ACME) and the U-Net-based deep learning baselines in terms of both overall accuracy and cell count accuracy. For the Ovules (plant) dataset, 3DCellSeg pipeline achieved performance comparable to the U-Net-based deep learning baselines that were initially built and fine-tuned based on the datasets of Arabidopsis thaliana cells, including the Ovules dataset. However, some of these U-Net-based deep learning baselines performed even worse than the ACME method on the HMS dataset (zebrafish cells), likely due to the use of extensive hyperparameters that are not transferrable across different cell types. In contrast, even without fine-tuning, 3DCellSeg still performed satisfactorily across the four different datasets because it has a simple structure and only utilizes one hyperparameter.

Moreover, 3DCellSeg is more robust than the object-detection-based deep learning baselines when the number of cell instances to be segmented is large. As compared to 3DCellSeg, when the number of cell instances was small on each slice (tens of cells), the object-detection-based deep learning baselines (including Mask R-CNN FPN and Mask R-CNN C4) achieved a higher accuracy on the LRP dataset. However, when the number of cell instances became large on each slice (around 100–300 cells), these object-detection-based baselines failed to accurately segment cells from the HMS and ATAS datasets.

Our ablation study reveals how the four components of the 3DCellSeg pipeline perform relative to their counterparts across four different datasets. Firstly, the use of 3DCellSeg Loss improved the segmentation accuracy of the original Dice Loss function for the four datasets, especially for the LRP and Ovules datasets with a higher cell shape irregularity. This is because 3DcellSeg Loss was designed specifically for cell segmentation and can suppress the cell foreground segmentation near the cell membranes where model confidence is not sufficiently high, thus reducing misclassification of voxels and enabling more accurate cell instance clustering.

Secondly, 3DCellSegNet slightly outperformed two CNN backbones commonly used in CT or MRI image segmentation (VoxResNet and 3D U-Net) for most performance metrics across the four datasets. This result suggests that a simple CNN structure with fewer parameters can fully capture the characteristics of cell membrane images that have a more recurrent structure than CT and MRI images. The simplified CNN structure can reduce the risk of model overfitting when limited training samples are available, thus providing a more robust backbone for cell segmentation across different datasets. In addition to improved accuracy, the light-weight design of 3DCellSegNet enables it to deliver much faster training and segmentation than the CNN models implemented in other pipelines. With 3DCellSegNet, a HMS image of 5 MB takes < 20 s to process, as compared to one of 30 MB or larger, which takes ~ 60 s to process, using VoxResNet or 3D U-Net.

Thirdly, the touching area-based clustering method, TASCAN, is slightly more accurate than the original clustering method DBSCAN. The marginal improvement in accuracy is probably due to the fact that most misclassified foreground masks have already been addressed by the 3DCellSeg Loss function in the first stage. However, TASCAN has a higher robustness and is easier to implement as it requires only one hyperparameter. In addition, the computational complexity of TASCAN is much lower because it processes super voxels rather than voxels. When dealing with larger images such as those in the LRP and Ovules datasets, TASCAN can significantly reduce the clustering time from one hour to a few minutes in the second stage.

Fourthly, the transfer learning experiment demonstrates the robustness of the whole 3DCellSeg pipeline across different datasets. When re-purposing a trained 3DCellSegNet model to segment cells in different cell membrane images, our 3DCellSeg pipeline still performs well, despite the fact that only a few new images are available for re-training.

Overall, as compared to other existing approaches to 3D cell segmentation, our approach is superior in three aspects: Firstly, our approach champions the use of domain-specific modelling to better capture the characteristics of the 3D cell images. We have utilized a light-weight CNN model with fewer parameters to perform semantic segmentation based on the knowledge that cell membrane images contain more recurrent patterns than CT or MRI images. Meanwhile, we have designed a custom loss function to address the misclassification challenge due to clumped cells. Secondly, our approach is robust and easy to fine-tune, requiring only one hyperparameter. Minimizing the number of hyperparameters is especially beneficial when adapting the pipeline to a new dataset, as the setting of hyperparameters is often not transferable across different datasets. Thirdly, the experimental results have demonstrated that our proposed method achieves performance better than or similar to state-of-the-art models but is more computationally efficient. They also suggest that our novel 3DCellSeg pipeline can accurately process high-throughput imaging data, allowing the automatic detection of 3D cells in a large scale in real-time, thus paving the way for cellular disease mechanism discovery.

Our work can be improved in two aspects. First, given that the CNN model is only used in semantic segmentation, we are yet to fully exploit deep learning models in learning representations of high-dimensional 3D images. In the future, we will build an end-to-end deep learning model to output the 3D cell instance segmentation directly. Second, despite the fact that we have accounted for the clumped cell issue, more domain-specific knowledge of the cell characteristics, such as the size and distribution of cells, can be integrated into our pipeline to improve cell identification/classification accuracy.

## Method

3DCellSeg performs semantic segmentation to identify the cell foreground masks, followed by instance segmentation. Two challenges of cell segmentation are addressed. The first is the clumped cell problem. Adhesions of the cell foreground masks need to be addressed during semantic segmentation, as they reduce the accuracy of instance segmentation. The second is the selection of hyperparameters, which is currently performed manually on a trial-and-error basis across a particular dataset, and may not be transferable across other datasets.

### Experimental setup

Consider a training set ${\mathcal{D}}$ of pairs $\left\{ {\left( {{\varvec{x}}_{i} ,{ }{\varvec{s}}_{i} } \right)} \right\}_{i = 1}^{N}$ where ${\varvec{x}}_{i}$ is a 3D cell membrane image and ${\varvec{s}}_{i}$ is the corresponding segmentation, where different integers represent different cell instances. Furthermore, consider a segmentation pipeline $h\left( f \right)_{\theta ,\lambda }$, where $f$ denotes semantic segmentation, $h$ denotes instance segmentation, $\theta$ denotes the CNN parameters to be trained, and $\lambda$ denotes the manually set hyperparameter(s).

### Dataset and metric

Four datasets containing 3D cell membrane images and corresponding voxel-wise cell labels were used for model training and testing. The first dataset, labelled as HMS, contains images of zebrafish cells. It is a new open-source dataset compiled by the Department of Systems Biology of Harvard Medical School. There are 36 images with a size of 181 × 331 × 160. A detailed description of HMS dataset is shown in the last subsection of Method. The second dataset, labelled as ATAS^49^, is an open-source dataset containing membrane images of Arabidopsis thaliana apical stem cells. It contains 126 cell membrane images with a size of 224 × 512 × 512. The third dataset, labelled as LRP^50^, is generated from three time-lapse videos documenting how Arabidopsis thaliana lateral root primordia developed. 27 images of the size of 2000 × 1000 × 500 are labelled voxel by voxel. The fourth dataset, labelled as Ovules^51^, contains 48 images of size 500 × 1000 × 1000 documenting all development stages of Arabidopsis thaliana ovules.

Our CNN model was trained for semantic segmentation, to generate the cell foreground, membrane, and background masks ${\varvec{g}}_{i}^{c}$ (every voxel in ${\varvec{g}}_{i}^{c}$ is 0 or 1; $c$ represents a category) for each image ${\varvec{x}}_{i}$. Additionally, to address the clumped cell problem, in the loss function we use a weight matrix ${\varvec{w}}_{i}^{c}$ that assigns a larger weight to voxels closer to the cell membranes. ${\varvec{w}}_{i}^{c}$ is calculated by applying a reverse distance transform^57^ to ${\varvec{g}}_{i}^{c}$. To reduce memory usage, we cropped ${\varvec{x}}_{i}$, ${\varvec{g}}_{i}^{c}$, and ${\varvec{w}}_{i}^{c}$ to small cuboids $\left\{ {{\varvec{x}}_{i}^{j} } \right\}$, $\left\{ {{\varvec{g}}_{i}^{c,j} } \right\}$, and $\left\{ {{\varvec{w}}_{i}^{c,j} } \right\}$, and trained the CNN model on cuboids $\left\{ {\left\{ {\left( {{\varvec{x}}_{i}^{j} ,{ }{\varvec{g}}_{i}^{c,j} ,{\varvec{w}}_{i}^{c,j} } \right)} \right\}_{j = 1}^{M} } \right\}_{i = 1}^{N}$.

We evaluated the performance of the segmentation based on four types of metrics: Jaccard Index (JI), Dice Similarity Coefficient (DSC), Adapted Rand Error (ARE), and Variation of Information (VOI). JI and DSC values were calculated for each cell, measuring the ratio of the correctly predicted voxels. We evaluated performance based on overall accuracy and cell count accuracy. Overall accuracy refers to the average values of JI and DSC of all cells (denoted as Avg JI and Avg DSC, respectively). Cell count accuracy is the fraction of cells whose JI (or DSC) is more than 0.7 (or 0.5). ARE measures how much the algorithm outperforms a random model^52^, and VOI is an entropy-based measure of clustering quality^53^. VOI_split_ measures split errors and VOI_merge_ measures merge errors.

1 $$JI\left( {seg,gt} \right) = \frac{{\left| {seg \cap gt} \right|}}{{\left| {seg \cup gt} \right|}}$$

2 $$DSC\left( {seg,gt} \right) = \frac{{2\left| {seg \cap gt} \right|}}{{\left| {seg} \right| + \left| {gt} \right|}}$$

3 $$ARE = 1 - maxFscore\left( {Rand\, Index} \right)$$

4 $$VOI\left( {seg,gt} \right) = 2H\left( {seg \cap gt} \right) - H\left( {seg} \right) - H\left( {gt} \right)$$

where $seg$ is the model prediction, $gt$ is the ground truth mask, and $H$ is the conditional entropy function. ${ }JI$ is the ratio of the intersection of $seg$ and $gt$ over the union of them while $DSC$ is the ratio of two times of the intersection over the sum of the size of $seg$ and $gt$. $ARE$ is derived from the Rand Index and $VOI$ is an entropy-based measure. Please see the complete definitions of $ARE$ and $VOI$ in^52^ and^53^ respectively.

### 3DCellSegNet for semantic segmentation

**Figure 4:**
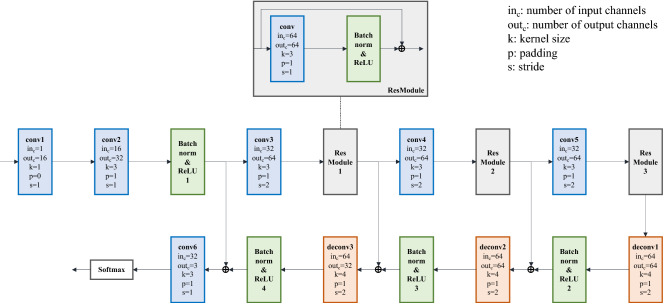
The structure of 3DCellSegNet. [Note: The extra voxels on the edge of the feature maps are removed after each deconvolution operation, in order to ensure the size of the up-sampled feature map is identical with that of the corresponding down-sampled feature map].

Figure 4 shows the structure of 3DCellSegNet. Based on the knowledge that cell membrane images contain more recurrent patterns than CT or MRI images, we built a lighter weight network for medical image processing which achieves faster inference speed with similar performance. For example, the average inference time of 3DCellSegNet on HMS and LRP is 20 and 50 s respectively, while the inference time of 3D U-Net and VoxResNet is around 2 times and 1.5 times longer. 3DCellSegNet has a shallow U-Net like shape with residual connection at the down sampling stage. Intuitively, for cell membrane images, only local features are required to determine the voxel category. In addition, by removing extra voxels on the edge of the feature maps during up-sampling, 3DCellSegNet can process cuboid-shape images of any size. We trained the model by feeding cuboids of slightly different sizes, thus improving the segmentation of cuboid interface and the model’s robustness.

We implemented 3DCellSegNet with PyTorch^58^. The model was trained on one Nvidia GeForce RTX 2080 Ti with a cuboid size ranging from 56 × 56 × 56 to 64 × 64 × 64. For the four datasets, the longest training time is 48 h, using Adam optimizer and a batch size of 7.

### 3DCellSeg Loss to tackle the clumped cell problem

Since cells are densely packed in the HMS dataset, there is an adhesion of the cell foreground masks in the semantic segmentation, thereby decreasing the accuracy of the instance segmentation. We proposed our 3DCellSeg Loss $L$ to tackle the problem.

5 $$L_{Others} = 1 - \frac{{2\mathop \sum \nolimits_{k = 1}^{K} p_{k} g_{k} w_{k} }}{{\mathop \sum \nolimits_{k = 1}^{K} p_{k}^{2} + \mathop \sum \nolimits_{k = 1}^{K} g_{k}^{2} }}$$

6 $$L_{Cell Foreground} = 1 - \frac{{2\mathop \sum \nolimits_{k = 1}^{K} \left( {\frac{{p_{k} }}{{p_{k} + \alpha }}} \right)g_{k} w_{k} }}{{\mathop \sum \nolimits_{k = 1}^{K} \left( {\frac{{p_{k} }}{{p_{k} + \alpha }}} \right)^{2} + \mathop \sum \nolimits_{k = 1}^{K} g_{k}^{2} }}$$

7 $$L = L_{Cell Foreground} + L_{Others}$$

where $p_{k} \in \left[ {0,1} \right]$ is the model confidence of a voxel being the cell foreground; $g_{k} \in \left\{ {0,1} \right\}$ is the voxel value of the category mask; $w_{k}$ is the weight of a voxel $k$ ($w_{k}$ is larger if $k$ is closer to cell membrane); $K$ is the number of voxels; $\alpha$ is a constant; $L_{Cell Foreground}$ is the loss function for the cell foreground and $L_{Others}$ is the loss function for other categories.

**Figure 5:**
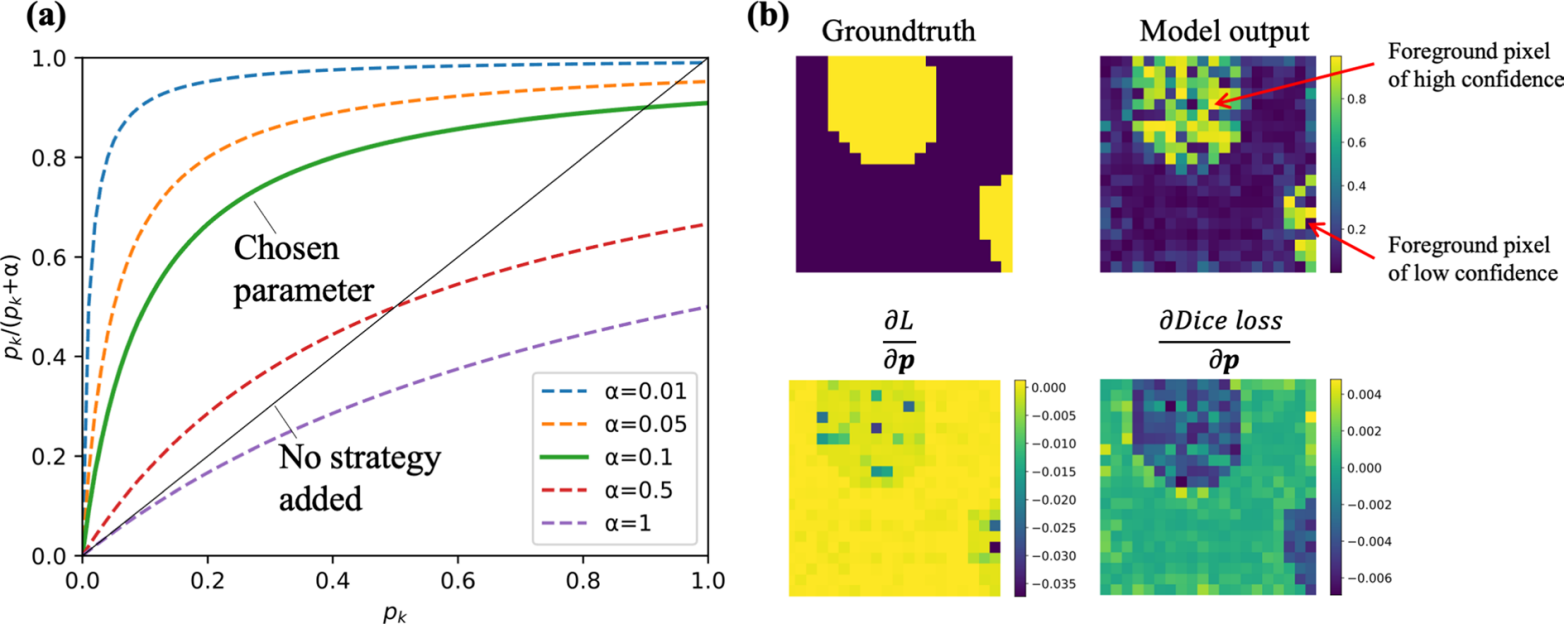
Addressing the clumped cell problem using 3DCellSeg Loss. [*Note* (**a**) $\frac{{p_{k} }}{{p_{k} + \alpha }}$ vs $p_{k}$ at different $\alpha$ values shows how different $\alpha$ affects the replacement $\frac{{p_{k} }}{{p_{k} + \alpha }}$. (**b**) A 2D slice of a simulation illustrating the difference between Dice Loss $\frac{{\partial L{ }}}{{\partial {\varvec{p}}}}$ and 3DCellSeg Loss $\frac{{\partial Dice Loss{ }}}{{\partial {\varvec{p}}}}$. The simulation results in (**b**) were generated by Python Matplotlib (https://matplotlib.org)].

We used weight matrix ${\varvec{w}}$ and replacement $\frac{{p_{k} }}{{p_{k} + \alpha }}$ to suppress the segmentation of the cell foreground close to the cell membrane if the confidence is not high, as the false positives of foreground voxels will result in clumped cell instances. 3DCellSeg Loss works like a regularizer, penalizing low confidence. Figure 5a shows the values of $\frac{{p_{k} }}{{p_{k} + \alpha }}$ at different $\alpha$ values. We chose 0.1 to achieve our desired regularization effect: when $p_{k}$ is small, the increasing rate of $\frac{{p_{k} }}{{p_{k} + \alpha }}$ should be high; when $p_{k}$ is near 0.5, $\frac{{p_{k} }}{{p_{k} + \alpha }}$ grows slowly as $p_{k}$ increases; however, even when $p_{k}$ is near 1, $\frac{{p_{k} }}{{p_{k} + \alpha }}$ should be slightly less than 1, thus maintaining the penalty.

$\frac{{\partial L{ }}}{{\partial {\varvec{p}}}}$ indicates that 3DCellSeg Loss penalizes heavily the positive voxels of low confidence (< 0.2), but penalizes slightly other positive voxels. $\frac{{\partial Dice Loss{ }}}{{\partial {\varvec{p}}}}$ penalizes the voxels on an equal basis. Hence, 3DCellSeg Loss will not push positive voxels of medium confidence (0.2—0.5) to a more positive end. The derivative of 3DCellSeg Loss $\frac{{\partial L{ }}}{{\partial p_{k} }}$ ($\frac{{\partial L{ }}}{{\partial p_{k} }} = \frac{{\partial L_{Cell Foreground} { }}}{{\partial p_{k} }} + \frac{{\partial L_{Others} { }}}{{\partial p_{k} }}$) provides a numerical explanation on how 3DCellSeg Loss works by comparing how $\frac{{\partial L{ }}}{{\partial p_{k} }}$ and $\frac{{\partial Dice Loss{ }}}{{\partial p_{k} }}$ change with $p_{k}$ in the top right box in Fig. 1.

8 $$\frac{{\partial L_{Others} { }}}{{\partial p_{k} }} = 2\frac{{ - g_{k} w_{k} \left( {\mathop \sum \nolimits_{l = 1}^{K} p_{l}^{2} + \mathop \sum \nolimits_{l = 1}^{K} g_{l}^{2} } \right) + 2p_{k} \mathop \sum \nolimits_{l = 1}^{K} p_{l} g_{l} w_{l} }}{{\left( {\mathop \sum \nolimits_{l = 1}^{K} p_{l}^{2} + \mathop \sum \nolimits_{l = 1}^{K} g_{l}^{2} } \right)^{2} }}$$

9 $$\frac{{\partial L_{Cell Foreground} }}{{\partial p_{k} }} = 2\frac{{ - g_{k} w_{k} \left( {\mathop \sum \nolimits_{l = 1}^{K} \left( {\frac{{p_{l} }}{{p_{l} + \alpha }}} \right)^{2} + \mathop \sum \nolimits_{l = 1}^{K} g_{l}^{2} } \right) + 2\left( {\frac{{p_{k} }}{{p_{k} + \alpha }}} \right)\mathop \sum \nolimits_{l = 1}^{K} \left( {\frac{{p_{l} }}{{p_{l} + \alpha }}} \right)g_{l} w_{l} }}{{\left( {\mathop \sum \nolimits_{l = 1}^{K} \left( {\frac{{p_{l} }}{{p_{l} + \alpha }}} \right)^{2} + \mathop \sum \nolimits_{l = 1}^{K} g_{l}^{2} } \right)^{2} }} \cdot \frac{\alpha }{{\left( {p_{k} + \alpha } \right)^{2} }}$$

In Fig. 1, the orange line indicates $\frac{{\partial L{ }}}{{\partial p_{k} }}$ while the blue line indicates $\frac{{\partial Dice{ }Loss{ }}}{{\partial p_{k} }}$. The two lines represent a simulation case where the model confidence of a cell foreground voxel $p_{k}$ varies from 0 to 1. As the figure shows, $\frac{{\partial Dice{ }Loss{ }}}{{\partial p_{k} }}$ follows a linear trend as $p_{k}$ changes. However, $\frac{{\partial L{ }}}{{\partial p_{k} }}$ imposes a heavy penalty for low confidence cases ($p_{k}$ < 0.2) but only a light penalty for high confidence cases (where the penalty is lower than $\frac{{\partial Dice{ }Loss{ }}}{{\partial p_{k} }}$). This shows that 3DCellSeg Loss works like a regularizer that penalizes model predictions of low confidence. The regularization effect of 3DCellSeg Loss is also illustrated in Fig. 5b which shows a 2D simulation. With the ground truth, where the yellow round shapes represent the cell foreground and the purple region represents the cell background, a simulated model output is generated. The lightness of the color indicates the confidence of a pixel being identified as a cell. Based on the ground truth and model output, the derivatives of Dice Loss and 3DCellSeg Loss over the 2D slice are shown in the images labelled $\frac{\partial L }{{\partial {\varvec{p}}}}$ and $\frac{{\partial Dice{ }Loss{ }}}{{\partial {\varvec{p}}}}$ respectively. Significantly, the mis-classified voxels around the edge are penalized more heavily than those in the center. Since mis-classification of the cell foreground often occurs near cell membranes, 3DCellSeg Loss helps address the adhesions of the cell foreground masks.

### TASCAN for instance segmentation

After the voxels were assigned different semantic labels, we applied Algorithm $h$ to the foreground voxels to divide them into different cell instances. The proposed TASCAN is inspired by DBSCAN^48^. However, our clustering algorithm is much more efficient. First, we applied watershed algorithm to pre-cluster the foreground voxels into super voxels, i.e., small clusters of voxels. Second, TASCAN was used to merge those super voxels that share a large touching area. The details of the algorithm are shown below.

Let $u$ be a super voxel. $S_{u}$ denotes the area of its surface. $V_{u}$ denotes the set of all super voxels that touches $u.$ For a super voxel $v \in V_{u}$, the touching area of $u$ and $v$ is denoted as $S_{uv}$. We also define a threshold value $minArea$. If the touching area of two super voxels is smaller than $minArea$, they are taken as two separate cells. We set the value of minArea at 30 for the four different datasets. This value is relatively robust and intuitively decided by the size of holes on the membrane (the area of the cell membrane that is mis-classified as the cell interior) in relation to the inference error generated by the backbone neural network. Basically, for an image with a much larger cell size, the value should be increased.

**Figure 6:**
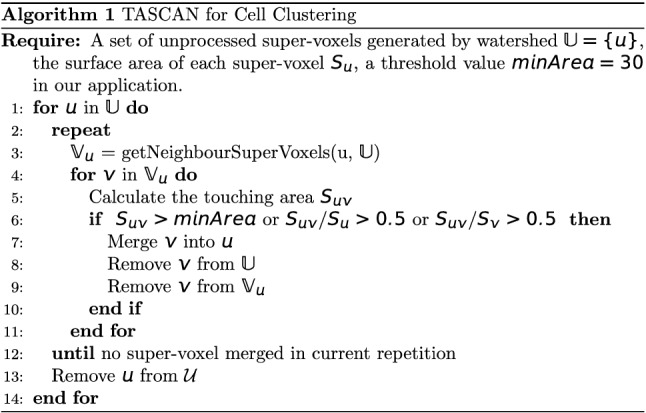
TASCAN algorithm for cell clustering.

Algorithm 1 (see Fig. 6) shows the operation of TASCAN. The watershed algorithm is used to generate small clusters within the normal cells. Small super voxels are merged with the surrounding super voxels if the touching area is greater than half of its surface area. The physical interpretation of setting the threshold to 0.5 is that if at least half of the surface of one voxel is surrounded by another voxel, these voxels will be merged. After TASCAN clustering has been performed, the unassigned foreground voxels are assigned to their nearest cells. In addition, the membrane voxels are also assigned to their nearest cells. Since the metric for judging whether cell $u$ and $v$ should be merged is symmetrical in terms of $u$ and $v$, TASCAN does not rely on the order of super voxels; the voxels in each experiment are processed in a random order.

TASCAN operates at a higher speed than DBSCAN. It is capable of processing a large number of voxels all at once, instead of processing each voxel individually. Additionally, TASCAN is good at separating clumped cells. Although our revised loss function can improve the accuracy of recognizing the cell membranes, membrane voxels can still be mis-classified as foreground pixels, since the foreground voxel clusters may be connected with each other. Whereas DBSCAN finds it difficult to separate these clusters if the search radius utilized is either too small or too large (exceeding the thickness of a membrane), TASCAN is able to resolve this problem. The clusters can mostly be separated firstly by the watershed algorithm, followed by separation performed by TASCAN as the connection tunnels are usually very thin.

### HMS dataset

Zebrafish transgenic embryos expressing nuclear-localized tomato and membrane-localized citrine (Tg(actb2:Hsa.H2B-tdTomato); Tg(actb2:mem-citrine)hm32,33), Tg(actb2:mem-citrine-citrine)hm30 were used to capture a time-lapse confocal dataset from 12–45 h postfertilization of development. High-resolution imaging was performed with Zeiss 710 confocal microscope with a Plan-Apochromat 40X 1.2 NA objective and a 514 nm laser (20mW, 3% laser power), pixel dwell time: 1.58 μs; pinhole size: 89 μm; line averaging: 1; image spacing: 0.2 × 0.2 μm, and 1024 × 1024 pixels per image, with an interval of 1.0 μm through Z for 80 μm, and temporal resolution of 2 min. A total of 225 timepoints were collected and 32 z-stacks were selected at regular intervals of 10 min for generating ground truth. The otic vesicle and inner lumenal surfaces were manually contoured in 2D using Gofigure2 and ITK-Snap. The surfaces were reconstructed in 3D using Powercrust reconstruction algorithm^59^. Since the otic vesicle is ellipsoidal, the images were rotated in 2D so that principal axes were aligned with coordinate axes. The 3D volumetric datasets were then cropped into a smaller size and resampled to have near isotropic sampling ratios (0.4 × 0.4 × 0.5 μm). To establish the ground truth dataset, cells were segmented using ACME. Semantic segmentation images were evaluated in 3D by overlaying on raw image data in GoFigure2 and stepping through z-stacks. Three types of segmentation errors were corrected. Missing cells, over- and under-segmented cells were identified, re-seeded, and the segmentations were re-generated. The generated segmentations were then evaluated manually a second time to guarantee highly accurate ground truth data. For example, the otic vesicle consists of epithelial cells that form a closed ellipsoid containing a fluid-filled lumen inside. This lumen is not a cell, so we manually relabelled the area as the background.

## Conclusion

Existing 3D cell segmentation methods face two challenges: (1) low accuracy in the presence of densely packed cells and (2) low robustness due to the need to fine-tune multiple manually selected hyperparameters on new datasets. This study aims to tackle these challenges by developing a deep learning-based two-stage pipeline, 3DCellSeg, for accurate and robust 3D cell segmentation with high computational efficiency. Specifically, the first stage utilizes a light-weight CNN model, 3DCellSegNet, to output the voxel-wise foreground, background, and membrane masks. A novel loss function, 3DCellSeg Loss, is incorporated into the model training process to address the clumped cell challenge. The second stage, which does not require any parameters from the first stage, pre-clusters the labeled voxels into super voxels using a standard watershed algorithm. A novel touching area-based clustering algorithm, TASCAN, is adopted to assemble the super voxels into cell instances while fine-tuning only one hyperparameter, i.e., the minimum touching area between two super voxels of the cell foreground, to better separate the clumped cells among the foreground voxel clusters.

Our experiments on four animal and plant datasets, namely, HMS (animal), ATAS (plant), LRP (plant), and Ovules (plant), show that 3DCellSeg has outperformed the state-of-the-art models on HMS, ATAS, and LRP, achieving an overall accuracy of 76.4%, 95.6%, and 74.7%, respectively. 3DCellSeg has also reached comparable performance to the state-of-the-art results from Ovules, achieving an overall accuracy of 82.2%. Our ablation studies further reveal that 3DCellSeg’s improvement in performance is attributable to 3DCellSeg Loss, 3DCellSegNet, and TASCAN, while showing why the whole 3DCellSeg pipeline is more robust across the four datasets. First, the use of 3DCellSeg Loss, a tailored loss function for cell segmentation, has improved the accuracy of voxel classification, especially for more irregular cells presented in the LRP and Ovules datasets. Second, 3DCellSegNet, a light-weight CNN structure using few parameters for cell segmentation, is more accurate and efficient than more complex CNN models when using different datasets with limited training samples. Third, TASCAN is slightly more accurate than its counterpart, is more robust and computationally efficient, requiring only one hyperparameter. Finally, after re-training 3DCellSegNet using a few samples from a different dataset, 3DCellSeg still performs well, demonstrating that our model is robust and transferable to new datasets. The experimental results and the ablation studies suggest that our novel 3DCellSeg can advance research on 3D instance segmentation; it can serve a powerful cell-based disease identification tool, such as cancer diagnostics, when our cell segmentation model is further trained on labelled human cancer/normal cell images. In the future, we will develop an end-to-end deep learning pipeline to segment cell instances in 3D images directly. We will also incorporate more domain-specific knowledge related to the cell characteristics into the pipeline to improve performance.

## Supplementary Information

Supplementary Information.

## Data Availability

The code is available on https://github.com/AntonotnaWang/3DCellSeg. The HMS dataset used during this study is available upon email request to the author Kishore R. Mosaliganti. The ATAS, LRP, and Ovules datasets used during this study are available from the previous studies cited in this article (Willis et al., 2016; Barro et al. 2019; Tofanelli et al., 2019).
