# Supplementary material for: A novel deep learning-based 3D cell segmentation framework for future image-based disease detection
Source: Sci Rep. 2022 Jan 10;12:342. doi: 10.1038/s41598-021-04048-3 (PMC8748745; doi:10.1038/s41598-021-04048-3)
Supplement: Supplementary file 1 — Supplementary Information. [file 41598_2021_4048_MOESM1_ESM.docx]

## Supplementary Information

**Traditional methods for cell segmentation:** Before deep learning becomes popular for image analysis, different techniques have been applied to cell segmentation. However, these methods have demonstrated a low segmentation accuracy and are labour-intensive. For instance, intensity thresholding methods generally fail when noisy images with clumped nucleus are used as the inputs, while marker-based watershed segmentation requires comprehensive handcrafting and precise parameter selection [9, 10]. ACME, a Hessian-based filtering method, is widely used as the baseline [3]. These methods have achieved low accuracy as compared to cutting-edge deep learning-based cell segmentation methods.

**Deep learning-based strategies for cell instance segmentation:** Deep learning-based approaches present a higher adaptability across different cell morphologies and staining patterns. Table 1 reviews the existing cutting-edge deep learning methods for cell segmentation and the drawbacks of each category of methods. A commonly deployed strategy is to first treat segmentation as a semantic segmentation task, and use deep learning methods to provide pixel-based prediction for cell interiors and edges, and backgrounds [4]. Clustering methods are then applied to generate predicted masks for different cells. We use the “contour-aware approach” to refer to any methods that use this type of segmentation strategy, because accurately classifying all contour pixels of cells is the foundation of instance segmentation in the second step. Convolutional Neural Networks (CNNs) have recently demonstrated state-of-the-art performance in cell semantic segmentation, and cutting-edge methods such as U-Net [5] and DeepCell [6]. Thresholding final probability map yields the final instance segmentation mask. Most commonly used methods in this step are thresholding and watershed [21, 22]. Deep Watershed [9] uses a CNN to learn a distance transform, producing markers for the follow-up watershed process. Conditional Random Field (CRF) can also be used in this step to improve the accuracy of generating the final instance masks [7].

We use the term “object detection-based” to refer to a new cell segmentation strategy. Corresponding to this strategy are methods including Retinanet [10], R-CNN and a series of revised structures [27–29], keypoint bounding box [14], and week annotation [15]. These methods are used to predict bounding boxes for all cells captured in an image, followed by generating a mask for each cell. Among all of these methods, Mask R-CNN achieves the highest accuracy [13], and has been widely applied in various segmentation tasks. [16] improves the general Mask R-CNN model with a weighted loss function for clustered cells, and [15] develops a 3D object detector to mask R-CNN to improve its performance utilizing semi-supervised training. [17] improves the mask generating process by making use of point-of-interest features, and achieves comparable accuracy with mush less time consumption. Some propose a one-stage objection detection model such as FCOS [18], similar to Mask R-CNN but without including the anchor proposal step. [19] proposes CenterMask, by adding a mask branch to FCOS, which can predict a mask for each identified box with a spatial attention map. YOLACT [20] also adopts this one-stage strategy and performs much faster than Mask R-CNN and achieves a dominating accuracy. The main drawback of these models is that they rely heavily on the setting of the number of anchor boxes, whose performance varies on different datasets. Also, they may fail to identify objects poorly approximated with bounding boxes.

In addition to the two strategies above, some deep learning methods focus on mapping the pixels of instances to a new space, where the pixels of different cells can be easily separated [34, 35]. These methods may not perform cell segmentation as accurate as their object-detection-based counterparts. This may be due to the spatial-invariant nature of the Fully Convolutional Networks [23]. Another method is to directly predict a suitable shape representation (star-convex polygon) for cell nucleus [24]. This approach takes on the assumption that the shape of the cells should be star-convex. A 3D-version of this approach has also been developed [25]. Generative Adversarial Networks (GAN) have been applied to cell segmentation [26], but the training process is highly complex, especially for 3D images. TensorMask introduces the sliding-window paradigm into instance segmentation, which generates bounding-box object predictions over a dense regular grid [27]. Instead of using generated bounding boxes as input, AdaptIS [28] and CondInst [29] perform instance segmentation by adapting themselves to the input point with the dynamic network layers, which produce different masks for different objects of the same image.

References

[1] F. Xing and L. Yang, “Robust nucleus/cell detection and segmentation in digital pathology and microscopy images: a comprehensive review,” *IEEE Rev. Biomed. Eng.*, vol. 9, pp. 234–263, 2016.

[2] X. Yang, H. Li, and X. Zhou, “Nuclei segmentation using marker-controlled watershed, tracking using mean-shift, and Kalman filter in time-lapse microscopy,” *IEEE Trans. Circuits Syst. I Regul. Pap.*, vol. 53, no. 11, pp. 2405–2414, 2006.

[3] K. R. Mosaliganti, R. R. Noche, F. Xiong, I. A. Swinburne, and S. G. Megason, “ACME: automated cell morphology extractor for comprehensive reconstruction of cell membranes,” *PLoS Comput Biol*, vol. 8, no. 12, p. e1002780, 2012.

[4] H. Chen, X. Qi, L. Yu, and P.-A. Heng, “DCAN: deep contour-aware networks for accurate gland segmentation,” in *Proceedings of the IEEE conference on Computer Vision and Pattern Recognition*, 2016, pp. 2487–2496.

[5] T. Falk *et al.*, “U-Net: deep learning for cell counting, detection, and morphometry,” *Nat. Methods*, vol. 16, no. 1, pp. 67–70, 2019.

[6] D. A. Van Valen *et al.*, “Deep learning automates the quantitative analysis of individual cells in live-cell imaging experiments,” *PLoS Comput. Biol.*, vol. 12, no. 11, p. e1005177, 2016.

[7] J. Jiang, P.-Y. Kao, S. A. Belteton, D. B. Szymanski, and B. S. Manjunath, “Accurate 3D Cell Segmentation Using Deep Features and CRF Refinement,” in *2019 IEEE International Conference on Image Processing (ICIP)*, 2019, pp. 1555–1559.

[8] D. Eschweiler, T. V Spina, R. C. Choudhury, E. Meyerowitz, A. Cunha, and J. Stegmaier, “CNN-based preprocessing to optimize watershed-based cell segmentation in 3D confocal microscopy images,” in *2019 IEEE 16th International Symposium on Biomedical Imaging (ISBI 2019)*, 2019, pp. 223–227.

[9] M. Bai and R. Urtasun, “Deep watershed transform for instance segmentation,” in *Proceedings of the IEEE Conference on Computer Vision and Pattern Recognition*, 2017, pp. 5221–5229.

[10] T.-Y. Lin, P. Goyal, R. Girshick, K. He, and P. Dollár, “Focal loss for dense object detection,” in *Proceedings of the IEEE international conference on computer vision*, 2017, pp. 2980–2988.

[11] R. Girshick, J. Donahue, T. Darrell, and J. Malik, “Rich feature hierarchies for accurate object detection and semantic segmentation,” in *Proceedings of the IEEE conference on computer vision and pattern recognition*, 2014, pp. 580–587.

[12] S. Ren, K. He, R. Girshick, and J. Sun, “Faster r-cnn: Towards real-time object detection with region proposal networks,” in *Advances in neural information processing systems*, 2015, pp. 91–99.

[13] K. He, G. Gkioxari, P. Dollár, and R. Girshick, “Mask r-cnn,” in *Proceedings of the IEEE international conference on computer vision*, 2017, pp. 2961–2969.

[14] J. Yi *et al.*, “Multi-scale cell instance segmentation with keypoint graph based bounding boxes,” in *International Conference on Medical Image Computing and Computer-Assisted Intervention*, 2019, pp. 369–377.

[15] Z. Zhao, L. Yang, H. Zheng, I. H. Guldner, S. Zhang, and D. Z. Chen, “Deep learning based instance segmentation in 3D biomedical images using weak annotation,” in *International Conference on Medical Image Computing and Computer-Assisted Intervention*, 2018, pp. 352–360.

[16] F. A. Guerrero-Pena, P. D. M. Fernandez, T. I. Ren, M. Yui, E. Rothenberg, and A. Cunha, “Multiclass weighted loss for instance segmentation of cluttered cells,” in *2018 25th IEEE International Conference on Image Processing (ICIP)*, 2018, pp. 2451–2455.

[17] L. Qi *et al.*, “Pointins: Point-based instance segmentation,” *IEEE Trans. Pattern Anal. Mach. Intell.*, 2021.

[18] Z. Tian, C. Shen, H. Chen, and T. He, “Fcos: Fully convolutional one-stage object detection,” in *Proceedings of the IEEE/CVF international conference on computer vision*, 2019, pp. 9627–9636.

[19] Y. Lee and J. Park, “Centermask: Real-time anchor-free instance segmentation,” in *Proceedings of the IEEE/CVF conference on computer vision and pattern recognition*, 2020, pp. 13906–13915.

[20] D. Bolya, C. Zhou, F. Xiao, and Y. J. Lee, “Yolact: Real-time instance segmentation,” in *Proceedings of the IEEE/CVF International Conference on Computer Vision*, 2019, pp. 9157–9166.

[21] C. Payer, D. Štern, T. Neff, H. Bischof, and M. Urschler, “Instance segmentation and tracking with cosine embeddings and recurrent hourglass networks,” in *International Conference on Medical Image Computing and Computer-Assisted Intervention*, 2018, pp. 3–11.

[22] Y. Xiang, C. Xie, A. Mousavian, and D. Fox, “Learning RGB-D feature embeddings for unseen object instance segmentation,” *arXiv Prepr. arXiv2007.15157*, 2020.

[23] R. Liu *et al.*, “An intriguing failing of convolutional neural networks and the coordconv solution,” *arXiv Prepr. arXiv1807.03247*, 2018.

[24] U. Schmidt, M. Weigert, C. Broaddus, and G. Myers, “Cell detection with star-convex polygons,” in *International Conference on Medical Image Computing and Computer-Assisted Intervention*, 2018, pp. 265–273.

[25] M. Weigert, U. Schmidt, R. Haase, K. Sugawara, and G. Myers, “Star-convex polyhedra for 3d object detection and segmentation in microscopy,” in *Proceedings of the IEEE/CVF Winter Conference on Applications of Computer Vision*, 2020, pp. 3666–3673.

[26] F. Mahmood *et al.*, “Deep Adversarial Training for Multi-Organ Nuclei Segmentation in Histopathology Images,” *IEEE Trans. Med. Imaging*, pp. 1–1, 2019.

[27] X. Chen, R. Girshick, K. He, and P. Dollár, “Tensormask: A foundation for dense object segmentation,” in *Proceedings of the IEEE/CVF International Conference on Computer Vision*, 2019, pp. 2061–2069.

[28] K. Sofiiuk, O. Barinova, and A. Konushin, “Adaptis: Adaptive instance selection network,” in *Proceedings of the IEEE/CVF International Conference on Computer Vision*, 2019, pp. 7355–7363.

[29] Z. Tian, C. Shen, and H. Chen, “Conditional convolutions for instance segmentation,” in *Computer Vision–ECCV 2020: 16th European Conference, Glasgow, UK, August 23–28, 2020, Proceedings, Part I 16*, 2020, pp. 282–298.
